# Telomere Fragment Induced Amnion Cell Senescence: A Contributor to Parturition?

**DOI:** 10.1371/journal.pone.0137188

**Published:** 2015-09-23

**Authors:** Jossimara Polettini, Faranak Behnia, Brandie D. Taylor, George R. Saade, Robert N. Taylor, Ramkumar Menon

**Affiliations:** 1 Division of Maternal-Fetal Medicine and Perinatal Research, Department of Obstetrics and Gynecology, The University of Texas Medical Branch at Galveston, Galveston, Texas, United States of America; 2 Department of Pathology, Botucatu Medical School, UNESP–Univ. Estadual Paulista, Botucatu, Sao Paulo, Brazil; 3 Department of Epidemiology & Biostatistics, Texas A&M University System Health Science Center, College Station, Texas, United States of America; 4 Department of Obstetrics and Gynecology, Wake Forest University, Winston Salem, North Carolina, United States of America; Shanghai Jiaotong University School of Medicine, CHINA

## Abstract

Oxidative stress (OS)-induced senescence of the amniochorion has been associated with parturition at term. We investigated whether telomere fragments shed into the amniotic fluid (AF) correlated with labor status and tested if exogenous telomere fragments (T-oligos) could induce human and murine amnion cell senescence. In a cross-sectional clinical study, AF telomere fragment concentrations quantitated by a validated real-time PCR assay were higher in women in labor at term compared to those not in labor. *In vitro* treatment of primary human amnion epithelial cells with 40 μM T-oligos ([TTAGGG]_2_) that mimic telomere fragments, activated p38MAPK, produced senescence-associated (SA) β-gal staining and increased interleukin (IL)-6 and IL-8 production compared to cells treated with complementary DNA sequences (Cont-oligos, [AATCCC]_2_). T-oligos injected into the uteri of pregnant CD1 mice on day 14 of gestation, led to increased p38MAPK, SA-β-gal (SA β-gal) staining in murine amniotic sacs and higher AF IL-8 levels on day 18, compared to saline treated controls. In summary, term labor AF samples had higher telomere fragments than term not in labor AF. *In vitro* and *in situ* telomere fragments increased human and murine amnion p38MAPK, senescence and inflammatory cytokines. We propose that telomere fragments released from senescent fetal cells are indicative of fetal cell aging. Based on our data, these telomere fragments cause oxidative stress associated damages to the term amniotic sac and force them to release other DAMPS, which, in turn, provide a sterile immune response that may be one of the many inflammatory signals required to initiate parturition at term.

## Introduction

Signals that initiate normal labor are still unclear [1] although multitudes of putative biochemical mediators and their pathways have been suggested as initiators [2,3]. The best documented signals occur in both maternal and fetal compartments and include endocrine (Corticotrophin relasing hormone [CRH], Adrenocorticotropic hormone [ACTH], functional progesterone withdrawal), immune (leukocyte and leukotriene activation) and mechanical factors (enhanced uterine stretching and amniochorionic membrane disruption). These factors cause an inflammatory activation (mostly mediated by cytokines), and prostaglandin production to transform a quiescent myometrium to an active contractile state at term [2,4–8]. Pathological activation of myometrial contractility by cytokines and prostaglandins also has been implicated in spontaneous preterm birth (PTB) [9–11]. Identification of the critical signals and understanding their molecular mechanisms that initiate parturition is essential for reducing the risk of PTB, a major pregnancy complication.

We have proposed that fetal signals to initiate parturition arise from senescent fetal membrane cells. Senescence is characterized by irreversible growth arrest of cells and is a mechanism associated with aging [12]. Senescence of fetal cells is a natural physiologic process that occurs throughout gestation [13] and is particularly noticeable at term [13,14]. Increased senescence is likely due to enhanced oxidative stress (OS) generated by the growing fetus, uterine stretch or other still unknown factors [14]. OS-induced damage to cellular elements causes structural and functional alterations, resulting in senescence [15]. Morphologic (enlarged cells, and round and swollen organelles) and biochemical features (senescence associated β-Galactosidase [SA β-Gal], of senescence are evident in fetal membranes from women in term labor compared to term not in labor [16]. AF from term labor also had dysregulated inflammatory markers compared to gestational age-matched not in labor samples, suggesting sterile inflammation (inflammation in the absence of infectious agent) and its associated senescence associated secretory phenotype (SASP), a unique set of inflammatory markers, that include cytokines, chemokines, growth factors, matrix degrading enzymes, inhibitors and various other agionists and antagonists [16]. We posit that the inflammatory milieu generated by senescent cells [17,18] activates functional progesterone withdrawal, produces uterotonins and signals parturition. *In vitro*, we recapitulated these findings in primary human amnion epithelial cells from term not in labor specimens. Amniotic epithelial cells exposed to OS developed SASP via activation of p38MAPK [19] resembling such changes seen in membranes from women in term labor [14]. Therefore, it is likely that senescence inducing risk factors of PTB cause pathologic activation of senescence and SASP that lead to preterm labor.

It has been suggested that increased levels of cell-free fetal DNA in the maternal circulation, released as a result of placental senescence, can activate parturition [20]. A potential source of cell-free DNA is from the telomeres, the chromosome end caps that stabilize the genome in humans and other long-lived mammals [21]. Accordingly, we have reported that telomere length reduction occurs in fetal compartments throughout gestation, with the shortest telomeres seen in term fetal membranes, suggesting a natural in utero aging process [13]. In human cells, telomeres range from 8,000–10,000 bp in length with a single-strand TTAGGG 3’ overhangs of 100–400 bases [22,23]. This terminal triplet of guanines is highly vulnerable to OS damage and single-strand breaks in this region are more resistant to nucleotide excision repair compared to the general genome [24]. The conversion of guanine into 8-oxoguanine (8-oxoG) is the most lethal OS induced lesion and the expectedly G-rich telomeres are highly susceptible to this damage [25]. We recently reported increased OS-induced DNA damage, predominated by 8-oxoG, due to reduced base excision repair by 8-oxoG glycosylase (OGG1) in human fetal membranes [26].

Previous data demonstrated that the majority of long-lived DNA damage foci in stress-induced senescent cells colocalize with telomeres, indicating that they are major contributors to a persistent DNA damage repair (DDR) mechanism [27]. Considering that telomere sequences are particularly vulnerable to OS [28], we tested the hypothesis that senescence of the fetal membranes, physiologically at term, results in telomere fragment release into amniotic fluid. Additionally, we demonstrated that oligonucleotides mimicking the telomere overhang sequence (T-oligos) activate cellular senescence and produce inflammatory cytokines through p38MAPK *in vitro* and *in situ* in human amnion epithelial cells and in pregnant murine models, respectively.

## Materials and Methods

### Institutional review board approval of the study

Amniotic fluid (AF) samples used for this study were from the Nashville Birth Cohort Biobank, established to study genetic and biomarker differences contributing to racial disparity in preterm birth. Samples were collected at Centennial Medical Center Nashville, TN, USA from 2008–2011. The study protocols for recruitment and collection of AF samples were approved by the Western Institutional Review Board, Seattle, WA; the reuse of samples for preterm birth related projects was approved by the Institutional Review Board (IRB) at The University of Texas Medical Branch (UTMB), Galveston, TX, USA. The authors complied with the World Medical Association Declaration of Helsinki regarding ethical conduct of research involving human subjects. Informed written consent was obtained from subjects prior to sample collection. Enrollment occurred at the time of admission for delivery.

### Subject recruitment and phenotype definitions

In this nested cross-sectional analysis, pregnant women between the ages of 18–40 years provided amniotic samples. Term specimens were obtained from women with a gestation age ≥ 37^0/7^ weeks; labor was defined as the presence of spontaneous, regular uterine contractions at a minimum frequency of 2 contraction/10 minutes, leading to delivery (term in labor group) and cervical dilatation. Women at term but not in labor (NIL) also were recruited. Details of this cohort and samples can be found in our other publications [29–33].

### Amniotic fluid sample collection

For vaginal deliveries, AF samples were collected during labor immediately before artificial rupture of the membranes by transvaginal amniocentesis of intact membranes using a 22 gauge needle through the dilated cervical os. In cases undergoing cesarean delivery, samples were collected by transabdominal amniocentesis. In order to isolate the telomere fragments from intact telomere repeat sequences from amniocytes and other cells in the AF, samples were immediately centrifuged three times at 3000 x g to remove all cells and particulate debris (amniotic sludge) [34] and supernatant aliquots were processed rapidly and stored in the dark at -80°C in filled tubes to minimize auto-oxidation during storage.

Demographic data were collected from patient interviews and clinical data were extracted from the patient medical records. Data collection included age, ethinicity, socioeconomic status (education, annual income and marital status), smoking, pre-pregnancy body mass index, and a complete medical and obstetrical history.

### Quantitation of telomere fragments in amniotic fluid

The final supernatants from stored AF samples were collected for DNA isolation using a commercial kit (DNeasy Blood and Tissue Kit, Qiagen, Germantown, MD) following the manufacturer’s recommendations. The quality and concentration of extracted DNA were determined by 260/280 nm absorbance ratio (Gen5, Epoch, Bio Tek, Winooski, VT, USA), and the relative concentration of telomere fragments was analyzed using quantitative real-time PCR (qPCR). References for relative number of telomere fragments were generated by performing serial dilutions from a reference DNA sample to produce concentrations of DNA ranging from 20 to 0.625 ng/μL. Quadruplicate (for standard curves) and triplicate (for samples) PCR reactions using 5 ng DNA for each sample were carried out in a 20μL volume using 2x DNA Master SYBR Green kit (Applied Biosystems (ABI), Foster City, CA, USA) on an ABI 7500 real-time PCR machine with SDS software, version 1.3.1. Primers for telomere (*tel1b*, 5'-CGG TTT GTT TGG GTT TGG GTT TGG GTT TGG GTT TGG GTT-3'; and *tel2b*, 5'-GGC TTG CCT TAC CCT TAC CCT TAC CCT TAC CCT TAC CCT-3') were added to the final concentration of 0.2 μM. The thermal cycling profiles were as follows: 95°C for 10 min, followed by 20 cycles of 95°C for 5 s, 56°C for 10 s, and 72°C for 60 s. Template controls were included in all plate reactions. The relative number of telomere fragments in each specimen was normalized to the reference sample [2^-(ΔCt(sample) – ΔCt(control)^ = 2^-ΔΔCt^] and β-Globin was used as for internal control gene.

### Primary human amnion cell cultures

#### Fetal membrane collection

Fetal membranes were dissected immediately after placental delivery from women undergoing elective repeat cesarean section for uncomplicated pregnancies at term, not in labor, at the John Sealy Hospital at UTMB, TX, USA. The IRB approval for discarded tissues was obtained prior to sample collection. The amnion layer was peeled from the underlying choriodecidua, washed in warm saline and small pieces (0.5 cm^2^) were digested twice with trypsin (1 mg/mL) and collagenase (0.5 mg/mL) for 30 minutes at 37°C. The digestion buffer was inactivated by DMEM complete media [(DMEM/F12 (Sigma-Aldrich, Saint Louis, MO, USA) supplemented with 15% fetal bovine serum (Sigma-Aldrich) and antibiotics (100 U/ml penicillin and 100 mg/ml streptomycin, (Sigma-Aldrich)] and the cells were collected by centrifugation. Cells were counted with a hemocytometer, and 1.5–2.0K cells were seeded in 10 cm culture flasks with DMEM complete media, at 37°C in a humidified atmosphere containing 5% CO_2_. The purity of the epithelial cells was greater than 95%, as determined by staining with cytokeratin antibodies (Pan-Cytokeratin, Abcam, Cambridge, MA, USA, #ab80826) [35,36]. The culture media were replaced every 48h. To control for effects of replicative senescence, all experiments were performed 8–10 days after primary culture. First passage cells were exposed to 40 μM telomere overhang mimetic sequence (T-oligos, [TTAGGG]_2_) or 40 μM control oligonucleotides (Cont-oligos, [AATCCC]_2_) for 48 hours. This working concentration was based on previously reported cytotoxic and/or cytostatic effects [37–40]. The oligonucleotides were purchased from Midland Certified Reagent Co. (Midland, TX, USA). Untreated cells were analyzed as a control. Results are representative from 5 independent cultures.

#### Cell viability assay

Cell viability was quantified based on a fluorescence assay. The membrane-impermeable dye propidium iodide (Sigma-Aldrich, #P4864) stains the nuclei of non-viable cells with red fluorescence, whereas the nuclei of all cells are stained with membrane-permeable Hoechst 33342 dye (Invitrogen, Carlsbad, CA, USA #H1399) [41]. Confluent cultures of amnion cells growing on glass chamber slides were evaluated for cell viability after 48h-treatment using 2.0 mg/ml propidium iodide and 1.0 mg/ml Hoechst 33342 for 20 minutes at 37°C under an Olympus BX43 fluorescence microscope with a URFL-T digital camera, and QCapture Pro software (Micropublisher 6.0, Burnaby, BC, Canada).

### Assessment of DNA damage

#### Immunofluorescence (IF) for phosphorylated (γ) histone H2AX

In order to evaluate the activation of the DNA damage response in amnion cells, we performed IF for phosphorylated histone γ-H2AX. Cells were fixed in ethanol 95% for 10 min at RT and blocked for 1h in PBS containing 1% BSA. Primary antibody (γ-H2AX, Abcam #ab22551) was diluted in blocking buffer and incubated for 3h. The cells were washed, incubated with secondary antibody (Dye Light 488, Abcam #ab96875) for 20min at RT, washed again, counter stained with 4’-6-diamidino-2-phenylindole (DAPI) and mounted with mount media. Images were acquired and analyzed under 40x magnification.

### Western Blot—p53, p38

Cultured amnion cells were lysed in RIPA lysis buffer with freshly added protease and phosphatase inhibitors (0.01%). The lysate was collected after scraping the culture plate and the insoluble material was removed by centrifugation at 10,000 rpm for 20 min at 4°C. The concentration of protein in each lysate was determined by using the BCA protein assay kit (Pierce BCA Protein Assay Kit, Thermo Scientific, Waltham, MA, USA). Equal protein (30 μg) from each sample was loaded onto a 10% SDS-PAGE gel and electrophoresed at 120 V. The resolved proteins were transferred to a PVDF membrane using the iBlot transfer apparatus (Bio-Rad Laboratories, Hercules, CA, USA). The membranes were blocked in Tris-Buffered Saline (TBS) containing 0.1% Tween 20 (TBS-T) and 5% skim milk for 2h at room temperature. Blots were incubated separately with antibodies against total p38MAPK (Cell Signaling, Danvers, MA, USA, #9212), phosphorylated (P)-p38MAPK (Cell Signaling, #9211S), p53 (Abcam, #ab1101), P-p53 (Abcam, #ab1431) or β-actin (Sigma-Aldrich, #A5441) at 4°C and shaken overnight. Blots were washed three times with TBS-T and incubated with appropriate peroxidase-conjugated IgG secondary antibody for 1h at RT. All blots were developed using chemiluminescence reagents ECL Western Blotting Detection System (Amersham Piscataway, NJ, USA), in accordance with the manufacturer’s recommendations, followed by autoradiography.

### Senescence-associated β-galactosidase (SA β-gal) assay

The SA β-gal activity, a senescent cell marker [42], was evaluated using a commercial histochemical staining assay, following the manufacturer’s instructions (Senescence Cells Histochemical Staining Kit; Sigma-Aldrich). Briefly, cells cultured in chamber slides were washed twice in PBS, fixed for 6–7 min with the provided Fixation Buffer, washed again in PBS and incubated for 1h at 37°C with fresh β-gal solution. Following incubation, cells were evaluated using a standard light microscope. The number of β-gal positive cells was scored by counting at least 300 cells per representative field and expressed as percentage of total cells [41].

### RNA isolation, cDNA preparation, and quantitative reverse transcription PCR

RNA was extracted from amnion cells using Direct-zol RNA Mini Prep kits (Zymo-Research, Irvine, CA, USA), according to the manufacturer’s instructions. The quality and concentration of extracted total RNA were measured by using Gen 5 Software, version 2.1 (Biotek Synergy H4 Hybrid Reader, Winooski, VT, USA) and the RNA samples (0.1 mg/mL) were subjected to reverse transcription using the High-Capacity cDNA Archive Kit (Applied Biosystems, Carlsbad, CA, USA), in accordance with the manufacturer’s instructions. The cDNA was used to quantify gene expression using TaqMan-validated primers and TaqMan MGB probes (Applied Biosystems) were used to amplify IL-6, IL-8, Toll like receptor (TLR)-9 and 16S (reference) genes (ID Hs00174131_m1, Hs00174103_m1, Hs00152973_m1 and Hs99999901_s1, respectively). The comparative 2^-ΔΔCt^ method was used for calculating relative gene expression.

### Luminex immunoassay for IL-6 and IL-8

Multiplex luminex-based immunoassays were performed for the cytokines IL-6 and IL-8 with the use of antibody-coated beads (Biosource International, Camarillo, CA, Luminex Corporation, Austin, TX, USA). Standard curves were developed with duplicate samples of known quantities of recombinant proteins that were provided by the manufacturer. Sample concentrations were determined by relating the samples absorbances to the standard curve by linear regression analysis. Concentrations below the assay detection limits (IL-6 = 5.89 pg/mL and IL-8 = 5.93 pg/mL) were considered as half of each value.

### Inhibition of p38MAPK induced senescence by SB203580 (p38MAPK inhibitor)

Considering the results regarding p38MAPK expression, additional experiments were performed using SB203580, a p38MAPK inhibitor, in order to verify the influence of p38MAPK activation on senescence profiles. Primary amnion cells were seeded for 24 hours before pretreatment with 30 μM SB203580 (Sigma-Aldrich, #S8307) for 6h. Subsequently, T-oligos, Control-oligos or complete media (untreated control) were added to the cells as described above. Senescence associated β-gal staining and IL-6 and IL-8 cytokine production were analyzed as described above.

### Confirmation of p53 inducibility in amnion epithelial cells

We verified the inducibility of p53 in primary amnion cells by treatment with 100 μM etoposide (Sigma-Aldrich, #E1383) for 24h. The drug was dissolved in 0.01% DMSO in DMEM complete media, and the control cells were treated with the same DMSO-media without the addition of etoposide [43]. Western blots were used to detect p53 expression.

### Murine model of telomere fragment exposure

To test induction of senescence by T-oligos, *in situ* studies were conducted using pregnant CD-1 mice (Charles River Laboratories, Wilmington, MA, USA). Animals were shipped on day 10 of gestation and acclimated in a temperature-and humidity-controlled facility with automatically controlled 12:12 hour light and dark cycles. Mice were allowed to consume regular chow and drinking solution ad libitum. The Institutional Animal Care and Use Committee (IACUC) at the University of Texas Medical Branch at Galveston, TX, USA approved the study protocol.

On day 14 of pregnancy, the mice (n = 5/group) were subjected to mini-laparotomy and each uterine horn was injected with saline (control), 60 nM T-oligo or Cont-oligo diluted in saline, either with or without 30 μM of SB 203580 in a final volume of 150uL. The T-oligo concentration was based on previous experimental data that demonstrated senescence in tumor cells after T-oligo injections [44]. Injections were done in between 2–3 gestational sacs (those most proximal to the cervix) on the left side of gravid uteri as previously described in the infection animal model [45]. Animals were allowed to recover in a warm environment and daily monitoring. We sacrificed the animals on day 18 by carbon dioxide inhalation according to the IACUC and American Veterinary Medical Association guidelines. Fetal weight, demise/absorption was recorded. Maternal serum, AF and amniotic sacs were collected from each animal and stored at -80°C. Amniotic sacs were analyzed regarding oxidative stress marker staining (3-nitrotyrosine modified proteins, 3-NT) by immunohistochemistry, p38MAPK activation by western blot and SA β-gal by specific immunostaining (as described above). Maternal serum and AF were analyzed for IL-6 and IL-8 by Luminex assay (as described above).

### Statistical analysis

#### Telomere fragments analyses

We categorized the clinical pregnancies into two outcome groups (term labor and term NIL). Comparisons between outcome groups and demographic and pregnancy characteristics were made using Pearson’s Chi-Square or Fisher’s exact tests when the cell size was less than 5. For continuous variables which were non-normally distributed, Mann-Whitney test was used to test for equality of the medians. For telomere analysis, telomere fragments were transformed to the square root. Means and confidence intervals were back-transformed for reporting. Representative means and standard deviation (SD) of amplifiable telomere fragments were assessed with t-tests and p < 0.05 was used for significance.

Based on group means, standard deviation and effect size (f = 0.46), a post-hoc power analysis revealed that we had >80% power for our t-test to detect a difference in amplifiable telomere fragments between groups at a 0.05 significant level. Square root transformation of the data was used due to the skewed distribution.

#### Data analysis from in vitro and in situ experiments

GraphPad Prism (version 5) software was used to calculate significant differences regarding densitometric quantitation of p38MAPK activation, percentage of SA β-gal positive cells and mRNA and protein expressions. ANOVA followed by Tukey's Multiple Comparison post-hoc test, or Kruskal-Wallis test, were used for comparison among the studied groups according to normal or non normal distributions, respectively.

## Results

### Clinical demographics

We compared demographic and clinical characteristics between 50 women in term labor and 51 women at term NIL. Women in term labor had a lower median maternal age, were less likely to be married and less likely to have a gravidity >2 when compared to term NIL, while no differences were seen in the other variables between the groups (Table 1). We used samples that are gestational age matched to assure that the effect we report in this study are not impacted by gestational age differences. Gestational age for term labor and term NIL women at delivery were, respectively, 39 (1.6) and 39 (0.8) (median, IQR); p = 0.55 (Table 1).

**Table 1:** Demographic, obstetric and clinical characteristics of studied patients according to the pregnancy outcome.

Variable	Term labor (n = 50)	Term not in labor (n = 51)	p value
** **Maternal Age Median (IQR)**	**25 (7)**	**29 (8)**	**0.0032**
**Black Race n (%)**			
**No**	**33 (67.4)**	**34 (68.0)**	**0.9446**
**Yes**	**16 (32.7)**	**16 (32.0)**	
**Missing n = 3**			
**Married n(%)**			
**No**	**24 (49.0)**	**12 (24.5)**	**0.0119**
**Yes**	**25 (51.0)**	**37 (75.5)**	
**Missing n = 5**			
**Educational Grade achieved n(%)**			
**< 12**	**6 (12.5)**	**2 (4.2)**	**0.2678**
**12**	**42 (87.5)**	**46 (95.8)**	
**Missing n = 7**			
**Unemployed n(%)**			
**No**	**26 (57.8)**	**20 (41.7)**	**0.1204**
**Yes**	**19 (42.2)**	**28 (58.3)**	
**Missing n = 11**			
**Income n(%)**			
**$50k+**	**8 (16.3)**	**14 (28.6)**	**0.0342**
**$30–50k**	**12 (25.0**	**19 (38.8)**	
**$15–30k**	**17 (34.7**	**6 (12.2)**	
**< $15k**	**12 (25.0)**	**10 (20.4)**	
**Missing n = 4**			
** **BMI**			
**Median(IQR)**	**25 (10.5)**	**27.5 (9.3)**	**0.1565**
**Missing n = 4**			
**Smoked n(%)**			
**No**	**43 (87.8**	**46 (93.9**	**0.4865**
**Yes**	**6 (12.2)**	**3 (6.1)**	
**Missing n = 3**			
**Gravidity n(%)**			
**<2**	**19 (40.3)**	**5 (10.6)**	**0.0009**
**2–5**	**28 (59.6)**	**42 (89.4)**	
**Missing n = 8**			
**Infant Sex n(%)**			
**Female**	**25 (52.1)**	**29 (63.0)**	**0.2827**
**Male**	**23 (47.9)**	**17 (37.0)**	
**Missing n = 7**			
**APGAR n(%)**			
**<7**	**1 (2.1)**	**3 (6.0)**	**0.2659**
**7–9**	**46 (97.9)**	**47 (94.0)**	
**Missing n = 4**			
** **GA at delivery (median, IQR)**	**39 (1.6)**	**39 (0.8)**	**0.5528**
** **Birth Weight (median, IQR)**	**3361.5 (572.5)**	**3355.0 (566.0)**	**0.8528**

P-values were derived by Pearson Chi-square test or Fishers exact.

**P-value for maternal age, body mass index, gestational age at delivery and birth weight were derived by Mann-Whitney test.

### Similarities and differences in telomere fragment levels

**Fig 1:**
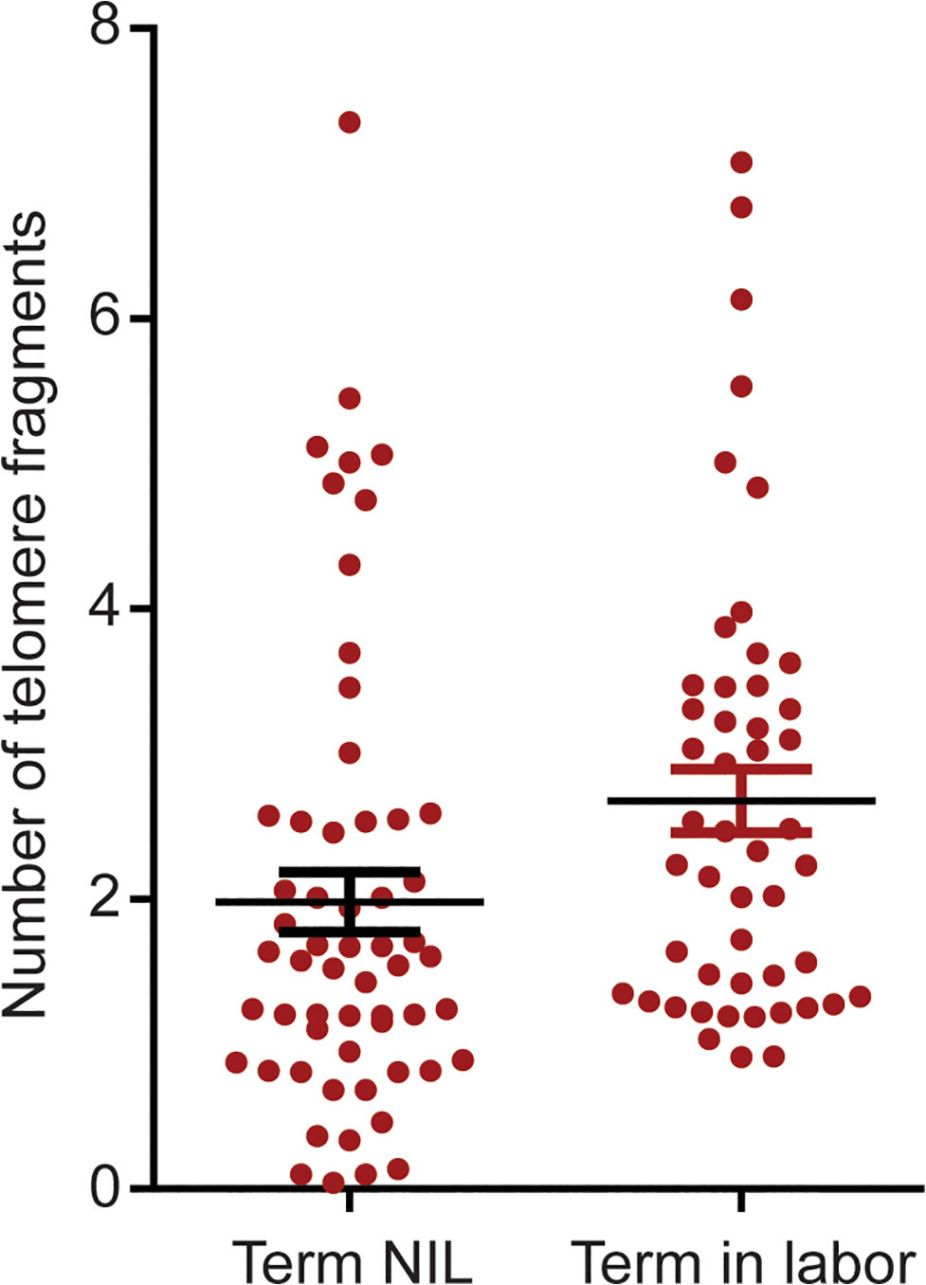
Quantitation of telomere fragments in human amniotic fluid. Scatter plot representing the number of telomere fragments detected in amniotic fluid from normal term not in labor (NIL) and term in labor samples. The distribution of telomere fragments significantly differs between groups (p = 0.04; t-test).

The mean levels of amplifiable AF telomere fragments were higher in term in labor than NIL [mean 2.4±0.2 (standard deviation SD) *vs*.mean 1.8±0.3; p = 0.04) (Fig 1).

### Telomere fragments are not cytotoxic to human amniotic epithelial cell cultures

**Fig 2:**
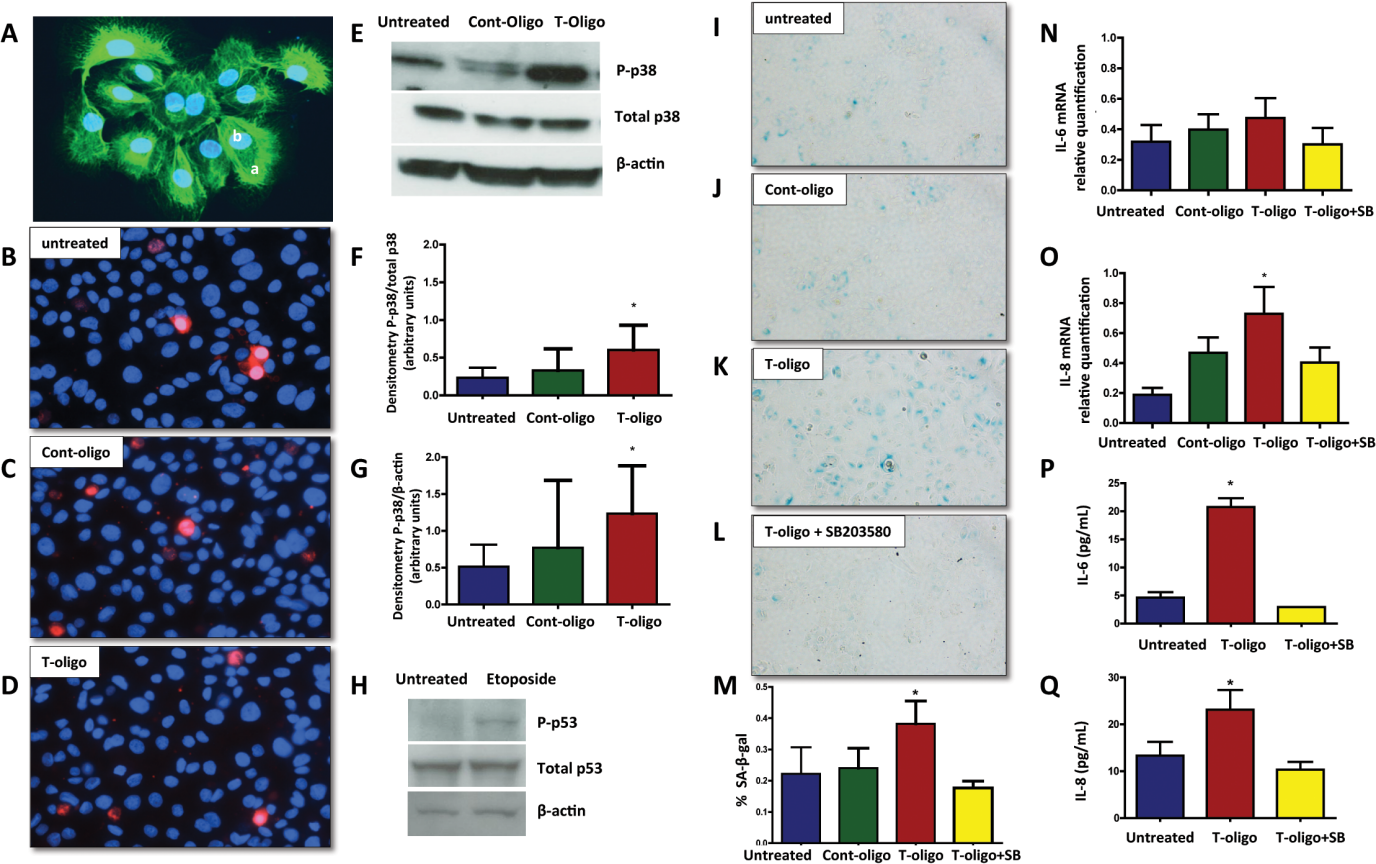
Human amniotic cells primary cultures. (A) Immunofluorescent staining of cytokeratin positive amnion epithelial cells. Inset, **a.** cytokeratin positive cells and **b.** nuclear staining DAPI. Original magnification x40. (B-D) Cell viability. Representative fluorescence photomicrographs of merged propidium iodide and Hoechst 33342-stained amnion cells. **B.** Untreated cells, **C.** Cont-oligo treated cells and **D.** T-oligo treated cells. Original magnification x40. (E-G) Representative image of Western blot analysis and densitometric quantitation of p38MAPK activation in amnion cells. E. Top panel = phosphorylated (P)-p38MAPK, middle panel = total p38MAPK and bottom panel = β-actin in untreated, Cont-oligo and T-oligo treated cells, respectively. F. Quantitation of P-p38MAPK densitometry normalized to total p38MAPK. T-oligo treatment produced significant (*) increase in P-p38MAPK compared to both untreated and Cont-oligo treated cells. G. Densitometric quantitation of P-p38MAPK normalized to β-actin. Post hoc tests indicated that T-oligo treatment produced significant (*) increase in P-p38MAPK compared to untreated control, but was not significant compared to Cont-oligo treatment. (*ANOVA, p<0.05). (H) Representative image of Western blot analysis of p53 activation in human amnion cells. Top panel = P-p53, middle panel = total p53 and bottom panel = β-actin in untreated and etoposide treated amnion cells, respectively. (I-M) Senescence associated β-galactosidase (SA-β-gal) staining of amnion cells. Single blue stained cells indicate positive β-gal activity. I. Untreated cells, J. Cont-oligo treated cells, K. T-oligo treated cells and L. T-oligo+SB203580 (p38MAPK inhibitor) treated cells. M. Quantification of the positive SA-β-gal cells. Bar graphs represent the differences in the percentage of SA-β-gal staining cells in each group. T-oligo treatment produced a significant increase (*) in the number of senescing cells, which was inhibited by SB203580 treatment. (*ANOVA, p<0.001). (N-Q) Senescence associated sterile inflammation in amnion cells. N. Relative quantification of IL-6 mRNA (p>0.05), **O.** Relative quantification of IL-8 mRNA in amnion cells (*p = 0.02), P. Protein concentration of IL-6 in conditioned media (*p<0.0001), and Q. Protein concentration of IL-8 in conditioned media (*p = 0.001). The production of IL-8 and IL-6 was inhibited by simultaneous treatment with SB203580. * ANOVA, T-oligo treated samples significantly higher compared with untreated, cont-oligo or T-oligo+SB samples. (Results are representative from 5 amnion cultures/ group. Telomere mimetic overhang sequence (T-oligo, [TTAGGG]_2_); control oligonucleotides (Cont-oligo, [AATCCC]_2_); untreated cells (Control CTR).

Concentrations of telomere fragments circulating in AF are higher under conditions that we previously documented to be associated with increased OS and short cellular telomeres, particularly term labor [13,14]. To test our hypothesis that telomere fragments might be toxic to primary human amnion epithelial cells (Fig 2A), we incubated synthesized telomere mimetic oligonucleotides (T-oligos), with amnion cells and evaluated their viability. As shown in Fig 2B–2D, there were no differences in propidium iodide exclusion (red staining) among untreated, Cont-oligo and T-oligo treated cells, confirming their viability after 48h in culture. We interpret these findings to indicate that the subsequent results reflect experimentally induced changes that are not originating from general loss of cell membrane integrity in culture.

### Amnion cell p38MAPK activation by T-oligo treatment

Previous reports from our lab indicated that amnion cells under oxidative stress develop senescence features primarily through p38MAPK activation [19], similar to those seen in human liver cancer cells [46]. As shown in Figs 2E–2G, T-oligo treatment induced higher P-p38MAPK than unstimulated controls (p = 0.02, ANOVA), but statistically significant differences were not achieved between T-oligo and Cont-oligo treated cells. Active (phosphorylated) p53 (P-p53) was not seen in amnion cells after treatment with T- or Cont-oligos. This raises a question of p53 inducibility in primary human amnion cells, as we have not seen p53 activation using any stimulants that cause OS and senescence. However, we did verify that p53 could be activated in these cells with 100 μM etoposide treatment for 24 h, a well-documented activator of the anticancer agent p53 tumor suppressor. (Fig 2H).

### T-oligos induce senescence phenotype and increase sterile inflammatory markers in human amnion epithelial cell cultures

Senescence was tested by SA β-gal staining after treatment with either T- or Cont-oligos. T-oligo treatment resulted in 1.7- and 1.6- fold increases in SA β-gal positive cells compared to Cont-oligo and untreated cells respectively (p = 0.004) (Fig 2I–2K). Although, we noticed some p38MAPK activation after Cont-oligo treatment, it did not result in development of senescence phenotype. To verify that senescence activation was mediated by p38MAPK, incubation with the p38 inhibitor, SB203580, was performed. As shown in Fig 2L, co-treatment with SB203580 decreased SA-β-gal positive cells compared to T-oligo treatment alone. The data are summarized in Fig 2M.

### Senescence associated secretory phenotype (SASP) activation

Inflammatory activation in senescing cells can modify the cellular environment. The expression of two inflammatory cytokines, interleukin (IL)-6 and IL-8, was studied in response to T-oligo treatment. A slight increase in IL-6 mRNA expression was noted, but did not reach statistical significance (Fig 2N), while IL-8 gene expression was significantly stimulated in T-oligo treated cells relative to Cont-oligo or untreated cultures (Fig 2O). Co-treatment with SB203580 significantly reduced IL-8 expression, confirming p38MAPK mediation. We further verified the release of IL-6 and IL-8 proteins from treated cells. Both cytokine levels were significantly higher following T-oligo treatment compared to all other groups, and levels in the conditioned media were reduced to untreated concentrations when co-incubated with SB203580 (Fig 2P and 2Q).

### Evidence of γ-H2AX formation

**Fig 3:**
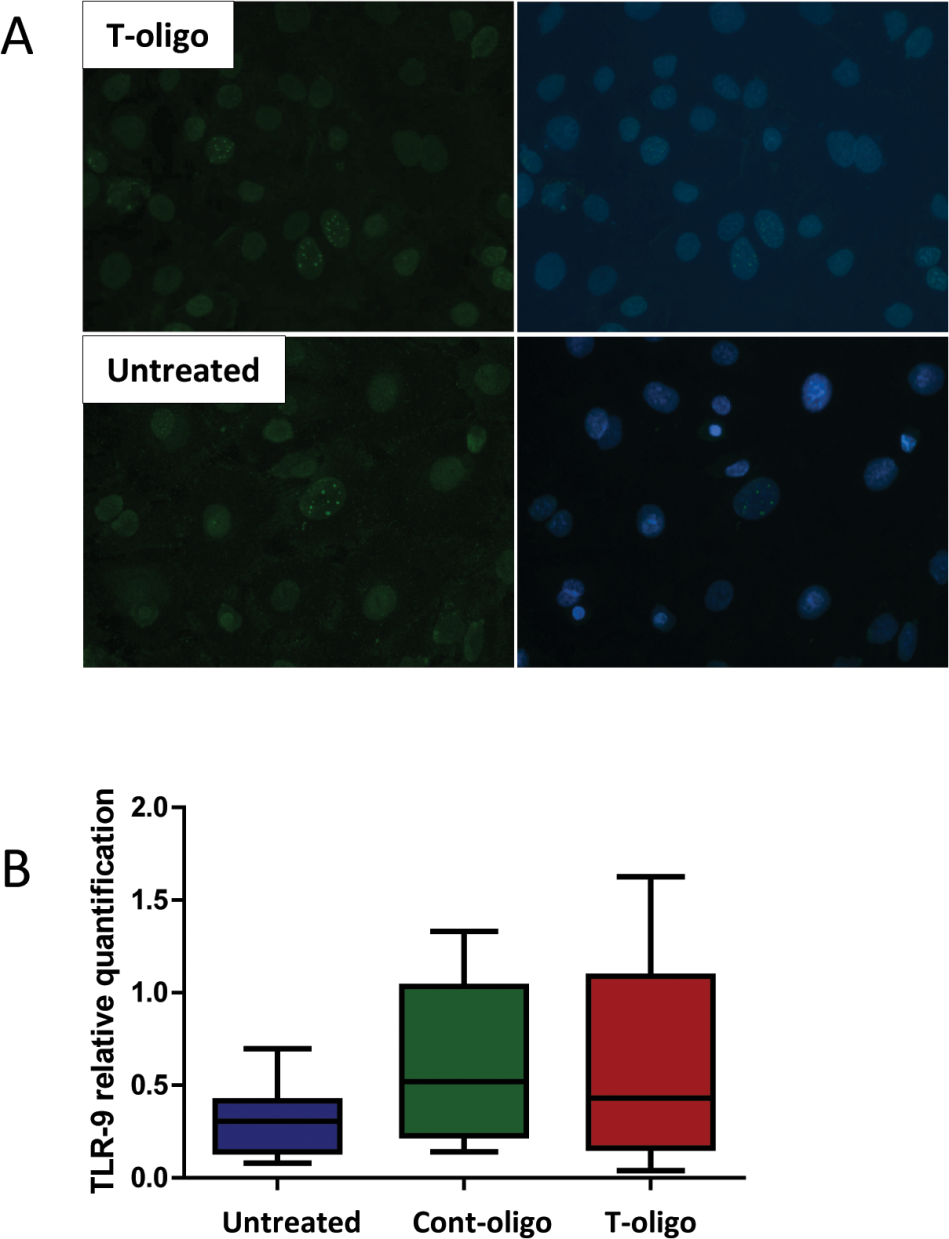
DNA damage foci and Toll like receptor (TLR)-9 expression in human amnion cells. (A) Immunofluorescence staining of phosphorylated (γ) H2AX, a marker for DNA damage response activation. Top panel = T-oligo treated amnion cells, bottom panel = untreated cells. Left panel = γH2AX. Right panel = merged images with DAPI nuclear stain. The bright nuclear dots represent DNA damage foci and are more pronounced in cells treated with T-oligo. (B) Relative quantification of TLR-9 mRNA expression in amnion cells in the studied groups, untreated cells, Cont-oligo and T-oligo treated cells, respectively. Box plots represent the quantification relative to endogenous 16S RNA. Kruskal-Wallis test, p>0.05.(control oligonucleotides (Cont-oligo, [AATCCC]_2_); Telomere mimetic overhang sequence (T-oligo, [TTAGGG]_2_).

With the purpose of demonstrating the activation of DNA damage repair, we performed immunofluorescence staining of phosphorylated (γ)-H2AX formation at so-called DNA damage foci (DDF). We found the DDF to be more pronounced in cells treated with T-oligo compared to untreated cells (Fig 3A), verifying more DNA repair activation.

### TLR-9 expression

In order to address a possible mechanistic pathway by which T-oligos activate intracellular signaling, we quantified TLR-9 mRNA. TLR-9 is known to trigger maternal immune cells activation in response to placenta-derived DNA [47]. However, we did not observe any difference in TLR-9 expression in amnion cells treated with T-oligos compared to controls (untreated and Cont-oligo samples) (Fig 3B).

### T-oligos induce fetal membrane senescence in pregnant mice

In order to validate our findings in an *in vivo* model, T-oligos were injected into the intrauterine compartment of pregnant CD-1 mice on day 14 of gestation. Specimens of amniotic fluid and amniotic sac were collected after sacrificing the dams on day 18. Table 2 depicts the descriptive general data regarding studied animals according to the treatment groups. There were no significant differences regarding placenta weight, animal or pup weight among the studied groups. The numbers of fetal demises and/or resorptions were significantly higher in T-oligo treated animals compared to vehicle (saline), Cont-oligo and T-oligo cotreatment with SB203580 (p = 0.001); however, preterm delivery was not observed in the studied animals. We cannot rule out a possible effect on late preterm delivery, as the injections were performed at ~70% of the colony gestational period and sacrifice was performed on day 18.

**Table 2:** Descriptive data from studied CD-1 pregnant mice according to treatments groups (n = 5 animals/group): Saline, Cont-Oligo, T-oligo and T-oligo co-treatment with SB203580.

	**Saline**	**Cont-oligo**	**T-oligo**	**T-oligo + SB203580**	P*
**Number of fetal demise and/or resorption sites**	1.66 (±0.57)	0.33 (±0.57)	3.33 (±1.15)*	0.60 (±0.54)	0.001
**Pup weight (g)**	1.26 (±0.14)	1.22 (±0.39)	1.41 (±0.98)	1.05 (±0.20)	0.87
**Placenta weight (g)**	0.11 (±0.02)	0.10 (±0.02)	0.16 (±0.01)	0.09 (±0.04)	0.25
**Animal weight Day 14 (g)**	42.93 (±4.35)	39.50 (±0.85)	42.14 (±1.15)	39.63 (±2.46)	0.21
**Animal weight Day 18 (g)**	51.34 (±7.93)	49.50 (±0.51)	47.47 (±9.5)	46.43 (±5.47)	0.77

(g: grams; SB203580: p38MAPK inhibitor)

*Anova, Tukey's Multiple Comparison Test, p = 0.001.

### Evidence of OS induction in murine fetal membranes by T-oligos

**Fig 4:**
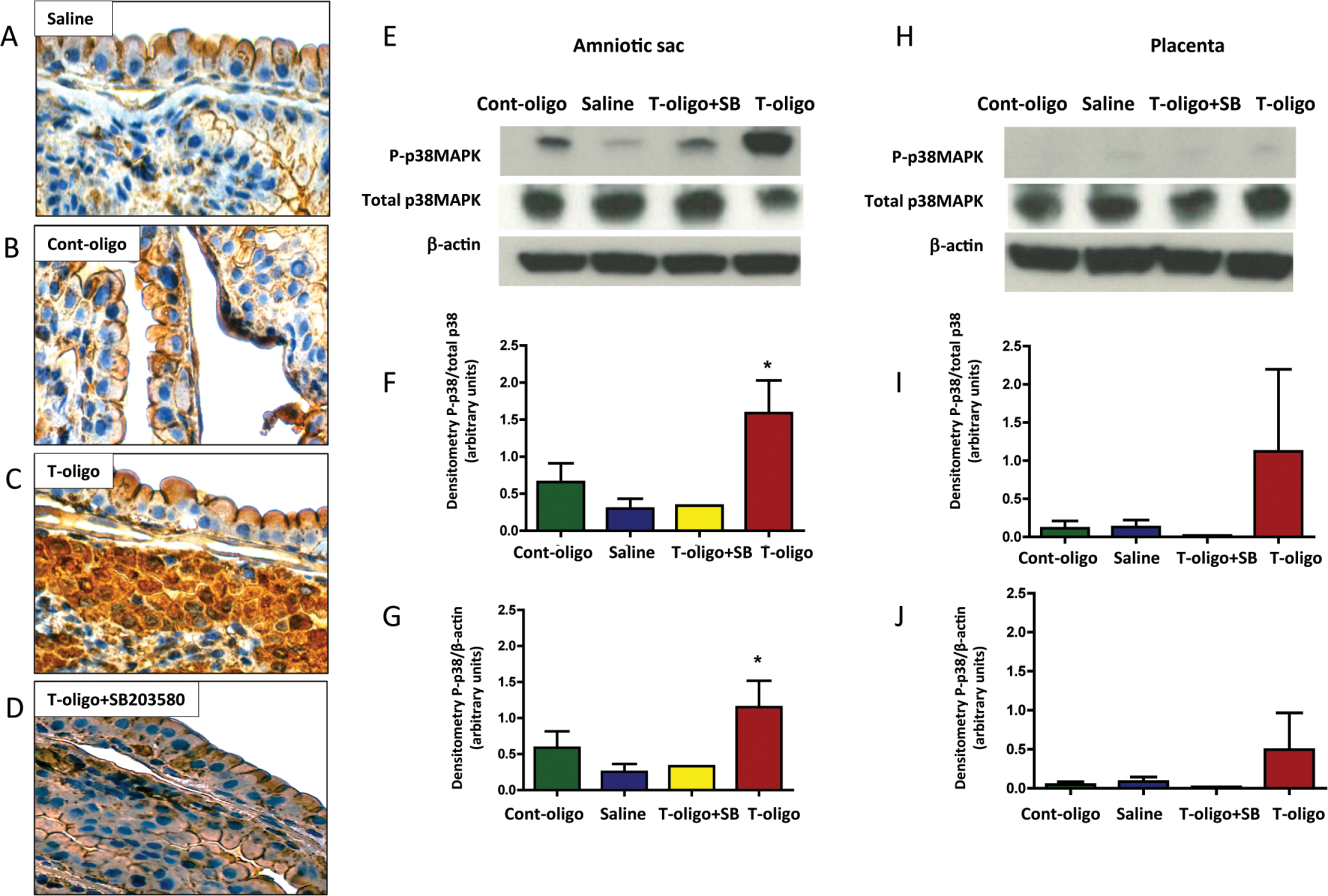
Animal models of T-oligo induced senescence. (A-D) Representative image of 3-nitrotyrosine (3-NT) modified proteins, an oxidative stress marker, in murine fetal membranes. Intrauterine injection of pregnant CD-1 mice were performed with either: A. Saline, B. Cont-oligo, C. T-oligo and D. T-oligo+SB23580 (p38MAPK inhibitor). **(**E-G) Representative image of Western blot analysis and densitometric quantitation of p38MAPK activation in murine amniotic sac. E. Top panel = phosphorylated (P)-p38MAPK, middle panel = total p38MAPK and bottom panel = β-actin in Cont-oligo, saline, T-oligo+SB203580 (p38MAPK inhibitor) and T-oligo treated mice, respectively. F. Densitometric quantitation P-p38MAPK normalized to total p38MAPK in amniotic sac tissue or G. normalized to β-actin. T-oligo treatment produced a significant (*) increase in P-p38MAPK compared to saline and Cont-oligo treated groups. The co-treatment of T-oligo and SB203580 showed similar results to controls (saline and Cont-oligo). (*ANOVA, p<0.05). Results are representative from 3 animals/ group. (Telomere mimetic overhang sequence (T-oligo, [TTAGGG]_2_); control oligonucleotides (Cont-oligo, [AATCCC]_2_).

In animals treated with vehicle (saline) or Cont-oligos, microscopic examination of the fetal membranes showed minimal evidence of OS, as expected in healthy metabolizing tissues (Fig 4A and 4B). However, we observed intense staining of 3-nitrotyrosine (3-NT) modified proteins confirming OS induced by T-oligos (Fig 4C). Co-treatment with T-oligos and SB203580 reduced the 3-NT staining intensity (Fig 4D).

### T-oligos activate p38MAPK in murine fetal membranes

As shown in Fig 4E–4G, T-oligo-injected mice showed increased P-p38MAPK in the amniotic sac compared to saline, which was reduced to control levels in animals simultaneously treated with SB203580.

### T-oligos cause senescence of murine amnion

**Fig 5:**
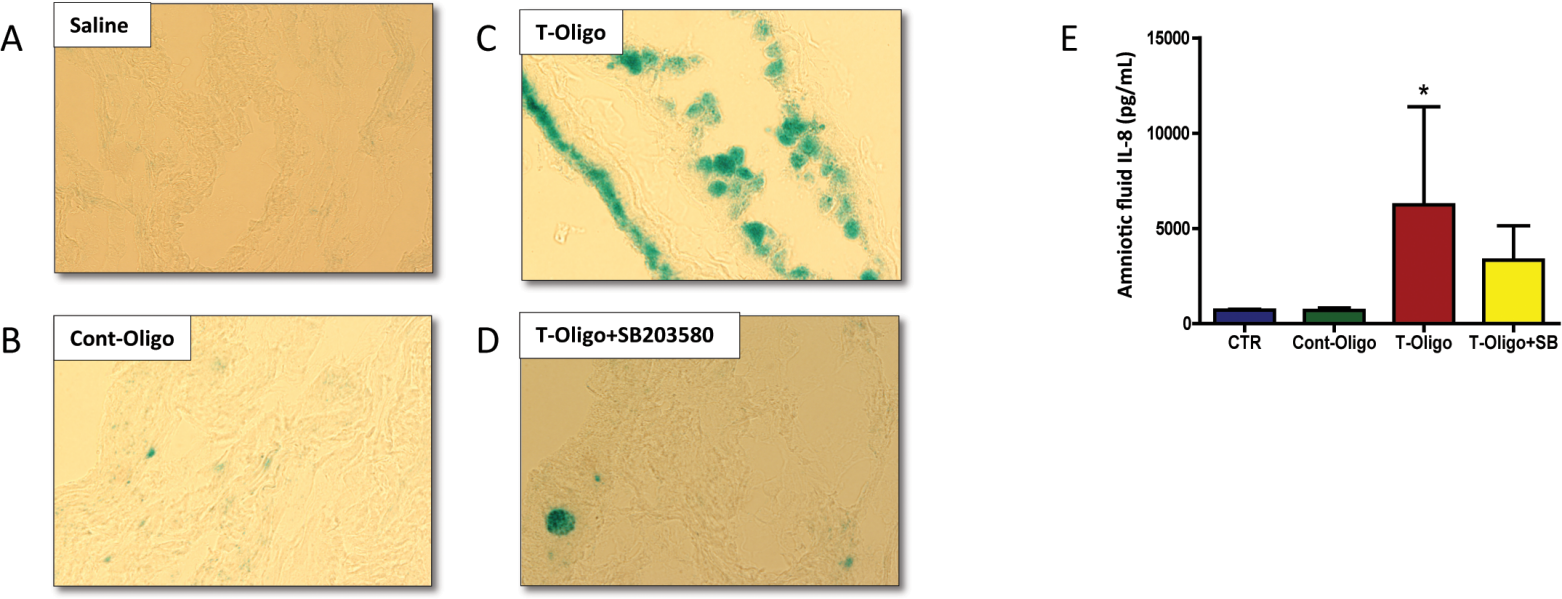
Senescence and inflammation induced by T-oligos in CD-1 pregnant mice. (A-D) Senescence associated β-galactosidase (SA-β-gal) staining of murine amniotic sac. Single blue stained cells indicate β-gal activity. A. saline, B. Cont-oligo, C. T-oligo and D. T-oligo+SB203580 (p38MAPK inhibitor) treated mice. SA-β-gal staining is pronounced in T-oligo treated mice. (E) Concentration of interleukin (IL)-8 protein in murine amniotic fluid. Higher levels of IL-8 were found in T-oligo treated animals compared to controls (saline and Cont-oligo). The production of IL-8 was inhibited by simultaneous treatment with SB203580. (*ANOVA, p<0.05). Results are representative from 3 animals/ group. (Telomere mimetic overhang sequence (T-oligo, [TTAGGG]_2_); control oligonucleotides (Cont-oligo, [AATCCC]_2_).

The senescence marker SA β-gal was evaluated histochemically in the mouse amniotic sacs. Intense blue staining representing senescent cells was observed particularly within the amnion epithelium of the membranes after T-oligo injection, compared to controls (saline and Cont-oligo) and T-oligo co-treatment with SB203580 (Fig 5A–5D).

### T-oligos increase biomarkers of sterile inflammation, indicators of SASP

The activation of sterile inflammation by T-oligos, as a result of amniotic sac senescence, was examined by measuring maternal serum and amniotic fluid cytokine levels in the mouse model. Amniotic fluid from animals injected with T-oligos manifested increased concentrations of all evaluated cytokines (IL-6 and IL-8), however, only IL-8 reached statistical significance (Fig 5E). Co-treatment with SB203580 decreased cytokine concentrations. No differences in cytokine levels were observed in maternal serum samples from the same dams.

## Discussion

The initiation of parturition is a complex process, whose precise signals remain unclear. Several systems maintain pregnancy through homeostatic balance including the endocrine, nervous, immune, hematological, microbiome, matrix metabolism and electro-physiological; antagonism of any of these tends to promote labor [2,7,9,48–50]. Breakdown of these balanced systems leads to cervical ripening, proteolysis, weakening and rupture of the fetal membranes and myometrial contraction leading to parturition [4,5]. Besides the well reported endocrine initiators, two general effectors of term parturition are oxidative stress and sterile inflammation, likely resulting from the augmented metabolic demands and depleted antioxidant reserves of the growing fetus [11,49,51]. This report opens a novel inquiry into the role of OS in the physiology of human parturition. The search for parturition triggers and prior data led us to hypothesize that OS at term signals fetal maturity and physiological aging of the fetal membranes, causing telomere shortening and sterile inflammation resulting in parturition [14,19,51]. This study provides insights into the telomere-dependent mechanism of senescence and inflammatory activation. The principal findings from this study are as follows: 1) The concentration of telomere fragments was higher in term labor than in term NIL samples. 2) T-oligos, that mimic shed telomeres, induced primary amnion epithelial cell senescence *in vitro* through the activation of p38MAPK and SASP, manifested by increased SA β-Gal and IL-8 gene and protein expression. Each of these steps can be mitigated using a p38MAPK inhibitor. 3) T-oligos caused murine fetal membrane OS, p38MAPK-mediated senescence and sterile inflammation (also reflected by elevated IL-8 concentrations). OS and cellular damage occur persistently during feto-placental growth [49,52,53], while cellular antioxidant proteins and repair responses prevent or minimize these damages, to avoid development of pathology. Diminished antioxidant capacity, or overwhelming OS, compromise tissue function and integrity and prompt aging [54]. Based on these principles, we propose a novel pathway whereby OS-induced DNA damage and telomere shortening in the fetal membranes accelerate senescence-associated inflammation, which acts as a fetal signal for parturition. In support of this hypothesis, OS related risk factors (e.g., cigarette smoking and low grade infection) contribute to telomere-dependent premature aging and inflammation as seen in a subset of early PTB and pPROM cases [51].

### Do telomere fragments trigger parturition?

The dose of telomere fragments used in our *in vitro* and *in situ* studies or design of our study did not demonstrate that telomere fragments can induce labor; instead, the telomere fragments presence is brought up as a possible stimulator of partiturion signals, since a feedback loop they cause oxidative stress associated damages to the term amniotic sac and forces them to release other Damage Associated Molecular Patterns (DAMPs) and SASP factors. We believe that this is one of the many mechanisms that can force amnion membrane to undergo further damage and send signals to the neighboring layers to initiate parturition process. These signals can force changes in decidua (activation of leukocytes) and myometrium (functional progesterone withdrawal). Telomere reduction is a natural consequence of repeated cell division [55]. Chronic OS increases the rate of telomere shortening and reduces a cell’s replicative life span [56,57]. Telomere shortening was previously observed in fetal leukocytes and placental membranes from term and pPROM pregnancies [13]. In line with this, here we demonstrate that more telomere fragments are shed into the amniotic fluid in term labor, compared to NIL subjects. *In vitro*, telomere shortening and replicative senescence can be accelerated using OS inducers like cigarette smoke [41], which exerts its effects through p38MAPK activation in amnion cells [19]. We suggest that the differences in cases of term labor *vs*. NIL status represents a buildup of OS mediators, inducing a senescent state. Premature senescence also can be induced by telomere-independent mechanisms [58].

Telomere shortening, sensed as DNA damage, can lead to further senescence by phosphorylation of histone H2AX (γ-H2AX), a highly conserved histone family member that encodes a DNA repair and transcription regulator [59,60]. To confirm that telomere fragments might induce DNA damage in isolated amnion cells, we tested the phosphorylation of H2AX. T-oligos activated DNA damage repair by γ-H2AX expression at so-called DNA damage foci (DDF) [61]. DDF, therefore, could be a surrogate for oxidative stress-induced telomere shortening in feto-placental tissues as previously reported in certain pathologic pregnancies [13,62,63].

DNA oligonucleotides homologous to the telomere 3′ overhang (T-oligos) have been studied in several cancer cell lines as a mechanism of cellular arrest and, consequently, represent new anti-cancer therapeutic opportunities [40,44,64,65]. However, their specific cytotoxic effects depend on cell type and environment. T-oligos induced senescence activation in human breast carcinoma [66], lung cancer [67] and prostate cancer [68] cells, while normal mammary epithelial cells [66] and human and murine lymphocytes [69] failed to show senescence under T-oligo treatment. Cell and tissue specificity are seen in pathways and responses [70]. Some senescent cells exhibit p53 activation [71], which has long been considered a key T-oligo signaling mechanism [37,39,72]. Senescence is triggered through multiple pathways; the p53 pathway in particular is known to accelerate aging in mammals [73,74]. During pregnancy, premature decidual senescence in mice with conditional deletion of maternal uterine p53 (p53^d/d^) was associated with PTB [75,76]. Moreover, cells from diverse tissue types lacking functional p53 undergo cell cycle arrest and senescence [69]. p53 activation was not evident in human amnion or murine fetal membrane cells exposed to T-oligos in our experiments; however, etoposide treatment verified that p53 is inducible in human amnion cells. Etoposide acts as pro-oxidant on intracellular thiols in cells, but the phenoxyl radicals formed from etoposide neither trigger phosphatidylserine (apoptosis-associated molecule) oxidation and externalization, nor do they induce lipid peroxidation. Instead, etoposide acts as an antioxidant against H_2_O_2_-induced phospholipid peroxidation in HL-60 cells [77,78]. These data indicate that the etoposide effect in our cells is not related to OS induction. Studies also show that p38MAPK activation represents an alternate senescence mechanism [46,65,79–81]. Intravenous administration of T-oligos rescued mice from a fatal inoculum of human breast cancer [66] and lung cancer cells treated with T-oligos showed reduced tumor volume through senescence pathways that are not dependent on p53 (56). Accordingly, our previous results indicated that p38MAPK triggers senescence in oxidatively stressed human fetal membranes and amnion epithelial cells [14,19]. Furthermore, attenuation of inflammatory cytokines after p38MAPK inhibitor treatment confirms the primacy of this pathway in the generation of sterile inflammation following T-oligo treatment in fetal cells and tissues.

The p38MAPK pathway is a major network of inflammation and stress responses [82] and in pregnancy, mediates IL-1β-induced MMP-9 in the fetal membranes [83], leading to labor. Recent findings from our laboratory reinforce the participation of p38MAPK in adverse pregnancy outcomes *in vitro* and *in vivo* [14,19]. Three p38 isoforms (α, χ and δ) activate distinct downstream cascades leading to DNA damage responses, whereby only p38δ is reported to be p53- and p16-independent [82]. However, the SB203580 inhibitor, which effectively reverses the senescence phenotype in our models, is believed to be selective for the p38 α and χ proteins. We did not explore the specific p38 isoforms expressed in amnion cells, hence we cannot discard the hypothesis that multiple p38δ isoforms might be activated in our cells.

Previous experiments using human breast carcinoma cells showed that T-oligos are efficiently taken up by cells within 30 to 60 minutes after *in vitro* administration and localize to the nucleus [66], where they are inherently more stable than non-G-quadruplex structures [84]. The Toll like receptor (TLR)-9 is a candidate receptor for T-oligo uptake, as it senses microbial DNA and endogenous cell-free-DNA [85,86]. However, we did not observe any difference in TLR-9 expression in amnion cells treated with T-oligos compared to controls (untreated and Cont-oligo samples). Thus, further experiments are needed to address the specific mechanism by which telomere fragments activate intracellular signaling in fetal cells.

Our data indicate that in addition to telomere shortening caused by ROS, the intracellular release of telomere fragments contributes to senescence of amnion cells via p38MAPK activation. The telomere fragments themselves can amplify fetal cell senescence triggering an inflammatory cytokine signature (SASP), which, in turn, can activate uterotonins and promote parturition. Activation of this axis prematurely, for example by excessive OS, may trigger preterm labor. A better understanding of the pathways activated by telomere fragments, their biochemistry and their contribution to fetal membrane senescence should contribute to the design of more effective labor assessment (perhaps including PTB risk), and direct diagnostic and therapeutic interventions for labor induction or prevention.
